# Cancer stem cell markers predict a poor prognosis in renal cell carcinoma: a meta-analysis

**DOI:** 10.18632/oncotarget.11672

**Published:** 2016-08-29

**Authors:** Bo Cheng, Guosheng Yang, Rui Jiang, Yong Cheng, Haifan Yang, Lijun Pei, Xiaofu Qiu

**Affiliations:** ^1^ Department of Urology, The Affiliated Hospital of Southwest Medical University, Luzhou 646000, China; ^2^ Southern Medical University, Guangzhou 510280, China; ^3^ Department of Urology, Guangdong No.2 Provincial People's Hospital, Guangzhou 510317, China

**Keywords:** renal cancer, prognosis, cancer stem cells, biomarker, meta-analysis

## Abstract

**Background:**

Relevant markers of CSCs may serve as prognostic biomarkers of RCC. However, their actual prognostic significance remains inconclusive. Thus, a meta-analysis was performed to reevaluate the association of CSCs-relevant markers (CXCR4, CD133, CD44, CD105) expression with RCC prognosis more precisely.

**Methods:**

PubMed and Embase were searched to look for eligible studies. The pooled hazard ratios (HR) with 95% confidence intervals (95% CI) were used to reassess the association of CSCs markers expression and RCC prognosis of overall survival (OS), cancer-specific survival (CSS), disease-free survival (DFS), and progression-free survival (PFS).

**Results:**

There were 25 relevant articles, encompassing 2673 RCC patients, eligible for meta-analysis. Overall pooled analysis suggested that high CSCs markers expression predicted poor OS (HR, 2.10, 95% CI: 1.73–2.55) and DFS (HR, 3.77, 95% CI: 2.30–6.19). High CXCR4 expression predicted worse OS (HR, 2.57, 95% CI: 1.95–3.40), CSS (HR,1.97, 95% CI: 1.50–2.59), and DFS (HR, 5.82, 95% CI: 3.01–11.25). CD44 over-expression correlated with a poor OS(HR,1.58, 95% CI: 1.14–2.18), CSS (HR, 2.58, 95% CI: 1.27–5.23), and DFS (HR, 4.49, 95% CI: 2.12–9.53) in RCC patients. CD133 was an independent favorable prognostic factor for CSS (HR, 0.4, 95% CI: 0.29–0.54).

**Conclusions:**

The presence of CSCs markers correlates with poor RCC outcome. CSCs may be potentially utilized as prognostic markers to stratify RCC patients, probably representing also a novel potential therapeutic target.

## INTRODUCTION

Renal cell carcinoma (RCC) is a significant health concern representing the ninth most common cancer worldwide [1]. It is estimated that there will be 62,700 new RCC cases and 14,240 deaths in the United States in 2016 [2]. Despite advances in RCC treatment and new developments in cancer surveillance, 25–30% of RCC patients present with advanced or distant metastatic disease and 20-40% develop recurrent disease after curative surgery [3, 4]. Metastatic RCC is notoriously resistant to chemotherapy and radiotherapy and thus the therapeutic options are limited. Although molecularly targeted therapies have revolutionized the treatment of these patients, acquired resistance to targeted therapies eventually ensues because of secondary mutation of the target protein and molecular alterations [5, 6]. Accordingly, the prognosis of metastatic RCC patients remains generally dismal and its 5-year survival rate is ∼10 percent [1].

Prognostic biomarkers are crucial to guide therapeutic options and surveillance strategies. TNM staging, nuclear grade, and histological subtype have been the most reliable prognostic factors [7]. However, the predictive accuracy remains limited due to individual variations [8]. Although some new prognostic and predictive markers have been identified, only a few biomarkers are used into practice [9]. Therefore, there is a great need to identify valid therapeutic and prognostic markers for tailoring therapy and follow-up.

Cancers are believed to be driven by a small subpopulation of cancer stem cells (CSCs), which are responsible for cell self renewal, multidifferentiation, tumor relapse, and progression [10]. Multiple lines of evidence have supported the existence of CSCs in RCC [10, 11]. RCC CSCs can be functionally identified by several cell surface markers including Prominin-1(CD133), CXC chemokine receptor 4 (CXCR4), CD44, and Endoglin (CD105) [12, 13]. Studies have investigated the role of RCC CSCs markers in prognosis. One group demonstrated that CD133 expression was not associated with clinical outcomes in RCC [14]. However, another concluded that expression of CD133 predicted favorable survival [12]. RCC is characterized by dysfunctional mutation of the von Hippel Lindau (VHL) gene, inactivation of which increases expression of CXCR4 [15]. Many studies have shown that CXCR4 was overexpressed in RCC and this predicted poor prognosis [16]. Downregulation of CXCR4 could be used as promising therapeutic option. Although CD44 expression exerted an unfavorable prognosis of RCC in one study [17], other studies have not confirmed this [18, 19]. CD105 has been identified as a RCC CSCs marker but it is unclear whether or not it is prognostic [20, 21]. Thus, it remains unclear which markers may be of value in determining prognosis.

Therefore, this meta-analysis was performed to determine the relationship between CSCs markers and clinical outcome of RCC. These results may provide more prognostic markers for RCC patients classification and surveillance and enable the development of CSC-targeted treatment strategies.

## RESULTS

### Search results and study characteristics

**Figure 1:**
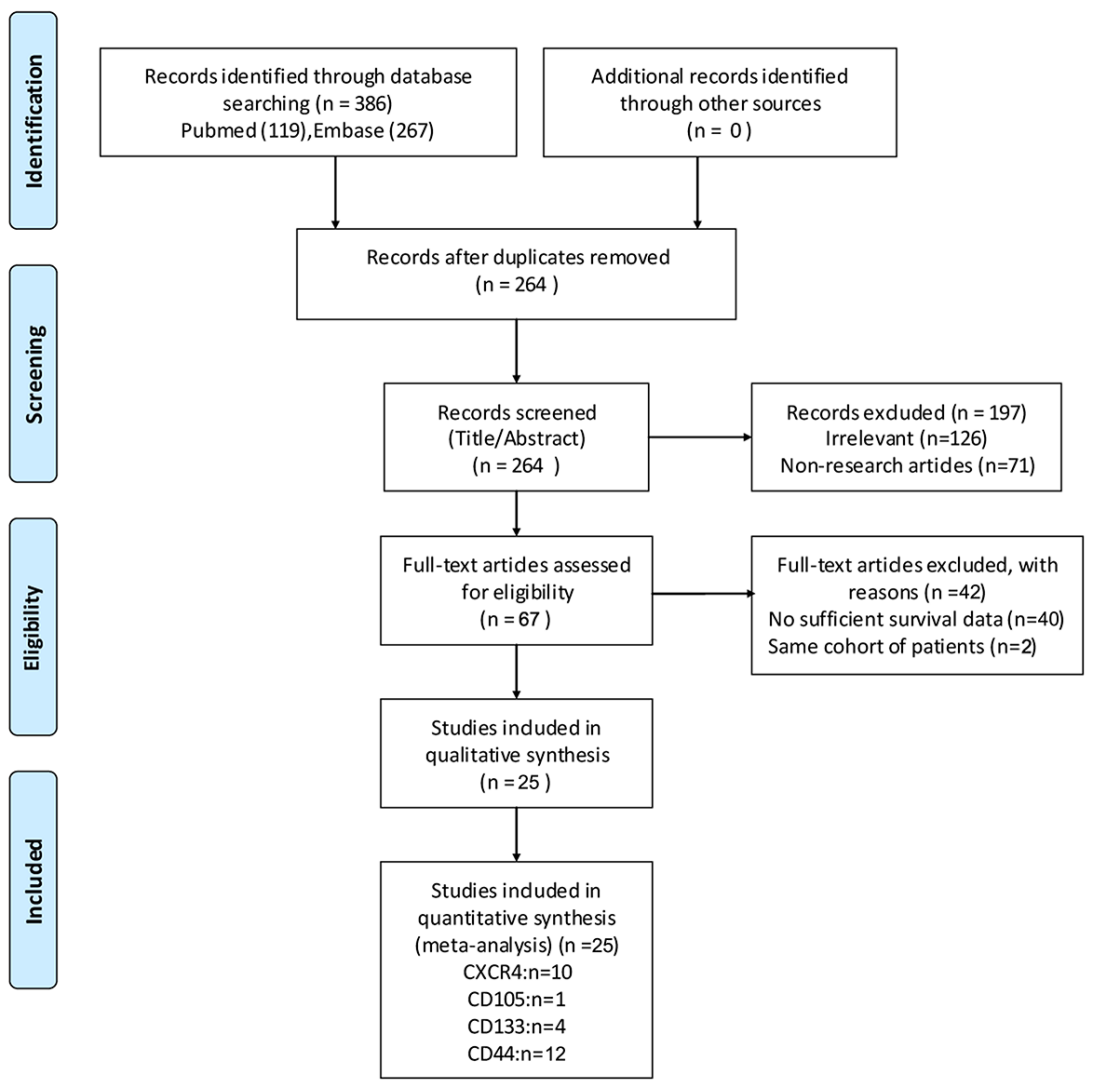
flow-chart of meta-analysis

The PRISMA flow diagram showing study selection procedure is shown in Figure 1. Cohen's kappa for inter-reviewer agreement was 0.81 (95% CI=0.77 to 0.85). After the initial database searches, 386 potentially relevant publications were identified. There were 319 studies excluded by assessing the title and abstract, including 122 duplicate reports, 126 irrelevant studies, and 71 non-research articles. A total of 67 remaining articles were further full-text reviewed, and then 42 papers were excluded because of insufficient survival information or duplicated cohorts. Finally, in accordance with the inclusion criteria, 25 articles [14, 15, 17-19, 22-41] about the association of CSCs markers expression and RCC survival were eligible for the meta-analysis.

The main characteristics of the 25 eligible studies are summarized in Table 1. These studies enrolled 2673 patients and were published between 1999 and 2014 with a median follow up of 54 months (range 22–85 months). All studies were retrospective cohort designs and the range of median age was 53- 64.5 years. Geographically, 10 studies were conducted in Asia, 12 in Europe, 1 in North America, and 2 in South America. There were 18 articles which reported HR and 95% CI directly, and the remaining studies were extrapolated and calculated from Kaplan–Meier curves. There were 21 studies which had their survival outcomes adjust for covariates (Supplemental Table S1). The CSCs markers expression was divided into positive and negative groups in all eligible studies. CSCs markers varied from different articles: 10 studies about CXCR4 expression [14, 15, 22-24, 26, 27, 30, 36, 41], 1 study about CD105 expression [35], 4 studies about CD133 expression [14, 29, 38, 40]], and 12 studies about CD44 expression [17-19, 25, 28, 31-34, 37-39]. According to NOS quality assessment, 14 studies were categorized as of high quality.

**Table 1:** Baseline characteristics of studies included in the meta-analysis

Author Year	Country Duration	Markers Pathological pattern	Sample size Median age	Treatment Detection method	Evaluation method	Cut-off level	Outcome indexes	Hazard ratios	95%CI	Multivariate analysis	Follow-up Mean/median (month)	Study quality#
D’Alterio et al 2010	Italy 1999-2007	CXCR4 RCC	240 61 (26-84)	SR IHC	Percentage	>20%	DFS	3.40	1.11–10.38	Yes	64	7
Huang et al 2014	China NR	CXCR4 RCC	45 57.7 (21-84)	NR TMA-IHC	CS	NR	OS	5.62	1.02-30.96*	No	NR	5
							DFS	6.89	1.21-39.23*			
Li et al 2011	China 2001-2005	CXCR4 LARCC	117 57.7 (31-82)	SR IHC	Intensity	NR	OS	4.12	1.79–9.47	Yes	51	8
D’Alterio et al 2012	Italy 2005-2009	CXCR4 mRCC	62 55	Sunitinib IHC	Percentage	>20%	PFS	2.04	1.08-3.84	Yes	29	6
							OS	1.48	0.93-2.38			
Li et al 2013	France 1999-2005	CXCR4 ccRCC	104 64.5 (34-86)	SR IHC	Percentage	>85%	CSS	2.60	1.11-6.10	Yes	79.5	7
							OS	2.20	1.11-4.38			
Wang et al 2012	China 2002-2003	CXCR4 RCC	97 55.4 (21-81)	SR TMA-IHC	Percentage	≥30%	DFS	8.03	3.19-20.22	Yes	NA	7
							OS	6.95	2.50-19.31			
Chen et al 2014	Germany 1992-2011	CXCR4 ccRCC/mRCC	44 NR	SR RT-PCR	NR	NR	CSS	3.8	1.1-13.9	No	NA	5
Staller et al 2003	Switzerland NR	CXCR4 ccRCC	195 NR	NR TMA-IHC	NR	NR	CSS	1.84	1.37-2.47*	No	NA	5
An et al (cohort 1) 2014	China 1996-2006	CXCR4 ccRCC	125 57.6	SR TMA-IHC	CS	>2	OS	3.38	1.49–7.68	Yes	62 (7-116)	9
An et al (cohort 2) 2014	China 1996-2006	CXCR4 ccRCC	100 60.5	SR TMA-IHC	CS	>2	OS	2.88	1.26–6.59	Yes	68(8–117)	9
Gassenmaier et al 2012	Germany NR	CXCR4 RCC/mRCC	88 NR	NC IHC	NR	NR	OS	4.1	1.2-14.8	Yes	NA	6
Saroufim et al 2014	France 2006-2009	CD105 ccRCC	102 62.2 (22-84)	SR IHC	Intensity	NR	OS	3.76	1.63–8.66	Yes	52(4-90)	8
							DFS	2.82	0.99–8.05			
Zhang et al 2013	China 1984-2008	CD133 mRCC	110 58 (36-76)	SR IHC	NR	NR	OS	1.59	0.84 – 2.99	Yes	64.71	8
Kim et al 2012	South Korea 1996-2008	CD133 pRCC	119 53 (11-75)	SR TMA-IHC	Percentage	NR	CSS	0.03	0.00-9.54	No	47.3(0.6-157.7)	7
Costa et al 2011	Brazil 1992-2009	CD133 RCC	142 54.7(23-81)	SR TMA-IHC	CS	NR	CSS	0.40	0.29-0.54*	Yes	NR	6
D’Alterio et al 2010	Italy 1999-2007	CD133 RCC	240 61 (26-84)	SR IHC	Percentage	>5%	DFS	1.26	0.55–2.87	Yes	64	7
Mikami et al 2014	Japan 1991-2003	CD44 ccRCC/mRCC	120 NR	SR IHC	Percentage	NR	OS	1.53	0.37 – 6.34	Yes	NR	8
Qin et al 2014	China 2006-2012	CD44 ccRCC	75 58.7 (29-82)	SR TMA-IHC	CS	NR	OS	2.67	0.83-8.61	No	52.6 (2-74)	9
Zhang et al 2013	China 1984-2008	CD44 mRCC	110 58 (36-76)	SR IHC	NR	NR	OS	1.46	0.82– 2.62	Yes	64.71	8
Costa et al 2012	Brazil 1992-2009	CD44 RCC/mRCC	99 55.5 (27-79)	SR TMA-IHC	CS	NR	CSS	1.11	0.39-3.18	Yes	NR	6
Tawfik et al 2007	USA 1995-2004	CD44 RCC/mRCC	62 61 (36-81)	SR IHC	CS	NR	OS	1.21	0.61-2.40*	Yes	22(0.1-108)	5
Lucin et al 2004	Croatia 1990-1998	CD44 RCC/mRCC	116 NR	NR IHC	Percentage	>25%	OS	3.25	0.93-11.35	Yes	85(1-165)	7
Yildiz et al 2004	Turkey 1988-1997	CD44 RCC	48 54 (20-82)	SR IHC	Percentage	NR	CSS	3.67	0.89-15.13*	No	48(1–168)	7
Bamias et al 2003	Greece 1996-1998	CD44 RCC	92 64 (46-86)	SR IHC	Percentage	>10%	OS	0.91	0.42-1.97*	No	41.5(30-65)	5
Rioux-Leclercq et al 2001	France 1992-1993	CD44 RCC	73 64 (37-86)	NR IHC	Percentage	NR	CSS	2.19	1.21-3.96*	Yes	52(9-75)	7
Daniel et al 2001	France 1987-1993	CD44 ccRCC	97 62.9 (37-85)	SR IHC	Percentage	NR	DFS	4.7	1.1–20.8	Yes	58.1(1-111)	8
Paradis et al 1999	France 1981-1990	CD44 ccRCC	91 58 (29-81)	SR IHC	Intensity	NR	DFS	2.89	1.5–5.2	Yes	54(1-38)	7
Jeong et al 2012	South Korea 2000-2006	CD44 ccRCC	110 60(30-78)	SR TMA-IHC	Intensity	>2	DFS	9.20	3.19–26.51	Yes	NR	6
							CSS	7.93	2.11–29.74			
							OS	4.00	1.44–11.12			

**Notes:** HR: Hazard ratio; OS: Overall survival; DFS: Disease free survival; PFS: Progression free survival; CSS: Cancer specific survival NR: Not reported; SR: Surgical Resection(radical nephrectomy or partial nephrectomy); IHC: Immunohistochemistry; CS: Complex score combining intensity and percentage; # Study quality was judged based on the Newcastle-Ottawa Scale (range, 1–9); *Estimated by survival curves. RCC: Renal cell carcinoma; ccRCC: Clear-cell renal cell carcinoma; mRCC: Metastatic renal cell carcinoma; LARCC: Locally advanced renal cell carcinoma; pRCC: Papillary renal cell carcinoma.

### CSCs markers expression and OS

**Figure 2:**
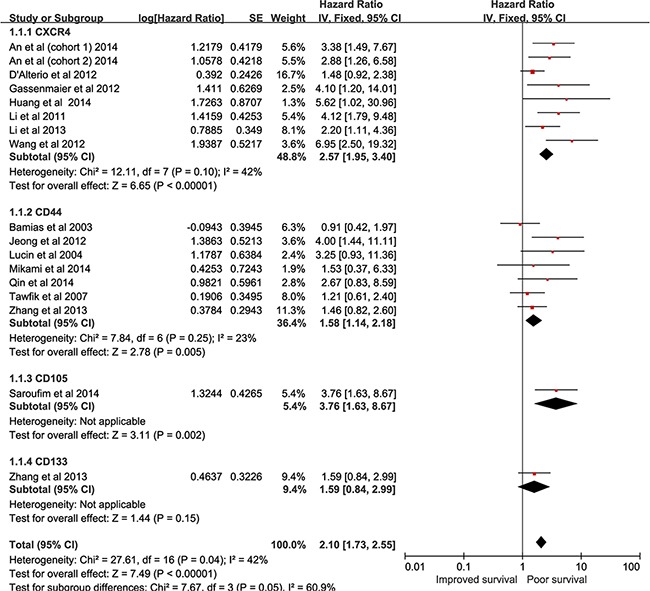
forest plot reflects HR with 95%CI for OS

A total of 1525 RCC patients from 16 studies included data for OS [17-19, 22, 24, 26-28, 30, 31, 33, 35, 36, 38, 41]. As shown in Figure 2, CSCs markers over-expression was significantly associated with poorer OS (pooled HR = 2.10, 95% CI = 1.73–2.55, P < 0.00001). The pooled data were not substantially heterogeneous (I^2^=42%); thus, a fixed-effect model was used. We investigated the association of individual CSCs markers with OS. High expression of CXCR4 (pooled HR = 2.57, 95% CI = 1.95–3.40, P < 0.00001) and CD44 (pooled HR = 1.58, 95% CI = 1.14–2.18, P =0.005) predicted worse OS. Limited articles reported the association of CD133 and CD105 with OS. One study [38] reported CD133 was not found to be a prognostic factor for OS using multivariate analysis (HR = 1.59, 95% CI = 0.84–2.99, P =0.15). Another study found that tumoral CD105 predicted poor OS (HR = 3.76, 95% CI = 1.63–8.67, P =0.002). Exploratory subgroup analyses were conducted according to study geography, sample size, study quality, disease stage, and HR origin. As shown in Table 2, these variables did not alter the prognostic role of CXCR4 in OS. Interestingly, the prognostic impact of CXCR4 was numerically higher in the Asia group (pooled HR = 3.97, 95% CI = 2.61–6.04, P < 0.00001) and high-quality studies group (pooled HR = 3.30, 95% CI = 2.29–4.76, P < 0.00001). Patients with CD44 high expression showed worse OS with respect to Asia (pooled HR = 1.97, 95% CI = 1.23–3.16, P =0.005), large sample size (pooled HR = 2.07, 95% CI = 1.23–3.48, P =0.006), and HR reported from study subgroup (pooled HR = 2.04, 95% CI = 1.35–3.08, P =0.0008).

**Table 2:** Subgroup analyses for OS and CSS

Outcomes	Subgroup	No. Of studies	No. Of patients	HR	95% CI		Effect Size		Heterogeneity	
							Z	P-value	P-value	I^2^
OS(CXCR4)	Geography									
	Asia	5	484	3.97	2.61	6.04	6.45	<0.00001	0.73	0%
	Europe	3	254	1.83	1.26	2.65	3.17	0.001	0.26	26%
	Sample size									
	Small (n <100)	4	292	2.23	1.50	3.31	3.97	<0.00001	0.02	69%
	Large (n >100)	4	446	2.96	2.00	4.38	5.43	<0.00001	0.70	0%
	Study quality									
	Low-quality	3	195	1.82	1.19	2.80	2.74	0.006	0.13	51%
	High-quality	5	543	3.30	2.29	4.76	6.40	<0.00001	0.44	0%
	Disease stage									
	Non-metastatic	5	471	2.32	2.18	4.80	5.81	<0.00001	0.43	0%
	Metastatic/mixed	3	267	2.06	1.39	3.40	3.61	0.0003	0.06	65%
	HR									
	Reported in study	7	693	2.52	1.90	3.34	6.42	<0.00001	0.08	47%
	Estimated from survival curves	1	45	5.62	1.02	30.96	1.98	0.05	-	-
OS(CD44)	Geography									
	Asia	4	415	1.97	1.23	3.16	2.81	0.005	0.35	8%
	Non-Asia	3	270	1.30	0.72	2.36	0.88	0.38	0.23	31%
	Sample size									
	Small (n <100)	3	229	1.25	0.75	2.08	0.87	0.39	0.32	12%
	Large (n >100)	4	456	2.07	1.23	3.48	2.73	0.006	0.31	17%
	Study quality									
	Low-quality	3	264	1.53	0.70	3.31	1.07	0.29	0.07	63%
	High-quality	4	421	1.78	1.13	2.81	2.50	0.01	0.6	0%
	Disease stage									
	Non-metastatic	4	387	1.75	0.96	3.18	1.84	0.07	0.11	50%
	Metastatic/mixed	3	298	1.52	0.87	2.65	1.49	0.14	0.4	0%
	HR									
	Reported in study	5	531	2.04	1.35	3.08	3.37	0.0008	0.43	0%
	Estimated from survival curves	2	154	1.07	0.64	1.78	0.25	0.8	0.59	0%
CSS(CD44)	Geography									
	Asia	1	110	7.93	2.11	27.95	3.07	0.002	-	-
	Europe	2	121	2.37	1.37	4.09	3.08	0.002	0.51	0%
	South America	1	99	1.11	0.39	3.18	0.20	0.84	–	-
	Sample size									
	Small (n <100)	3	220	2.01	1.24	3.27	2.83	0.005	0.37	0%
	Large (n >100)	1	110	7.93	2.11	27.95	3.07	0.002	-	-
	Study quality									
	Low-quality	2	209	2.85	0.42	19.47	1.07	0.29	0.02	81%
	High-quality	2	121	2.37	1.37	4.09	3.08	0.002	0.51	0%
	Disease stage									
	Non-metastatic	3	387	3.31	1.55	7.07	3.09	0.002	0.2	37%
	Metastatic/mixed	1	99	1.11	0.39	3.18	0.20	0.84	–	-
	HR									
	Reported in study	2	209	2.85	0.42	19.47	1.07	0.29	0.02	81%
	Estimated from survival curves	2	121	2.37	1.37	4.09	3.08	0.002	0.51	0%

### CSCs markers expression and CSS

**Figure 3:**
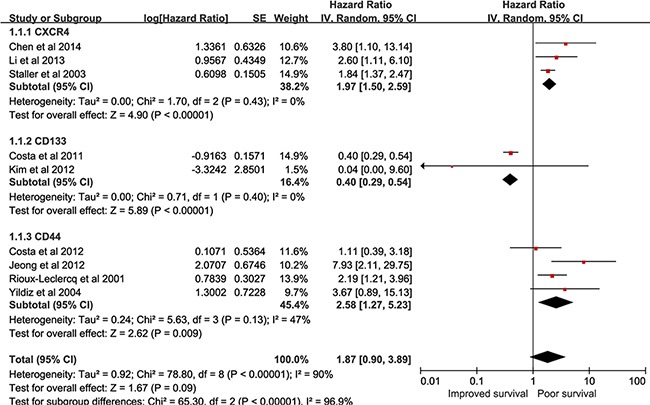
forest plot reflects HR with 95%CI for CSS

Nine studies comprising 934 patients reported the association of CSCs markers expression with CSS [15, 23, 28, 29, 34, 37, 39-41]. As shown in Figure 3, overall analysis suggested that high expression of CSCs markers was not linked to CSS (pooled HR = 1.87, 95% CI = 0.90–3.89, P =0.09). Furthermore, high CXCR4 expression was significantly related to poor CSS (pooled HR = 1.97, 95% CI = 1.50–2.59, P < 0.00001). RCC patients possessing high CD133 expression improved CSS (pooled HR = 0.4, 95% CI = 0.29–0.54, P < 0.00001). There was a significant association between enhanced CD44 expression and CSS (pooled HR = 2.58, 95% CI = 1.27–5.23, P =0.009). Subgroup analyses were carried out to explore heterogeneity. As shown in Table 2, results revealed that CD44 expression was not associated with CSS in low-quality studies group (pooled HR = 2.85, 95% CI = 0.42–19.47, P = 0.29, I^2^=81%).

### CSCs markers expression and DFS

**Figure 4:**
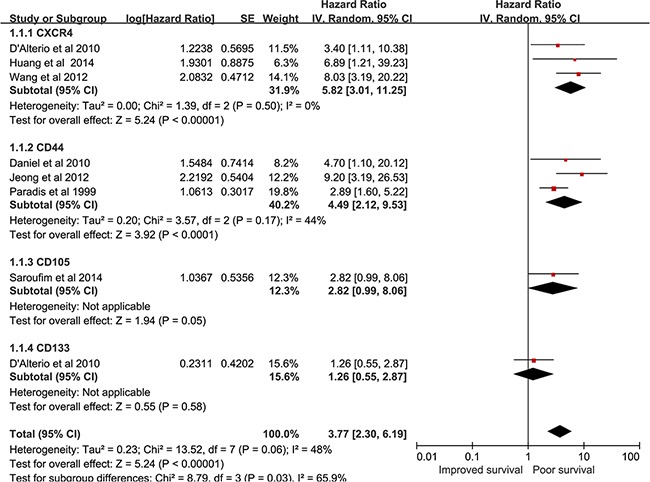
forest plot reflects HR with 95%CI for DFS

Eight studies encompassing 1022 patients assessed the relationship between CSCs markers expression and DFS [14, 25, 27, 28, 32, 35, 36]. As seen in Figure 4, overall, the adverse prognosis effect of high CSCs markers expression on DFS was seen (pooled HR = 3.77, 95% CI = 2.30–6.19, P < 0.00001). A combined analysis showed that high CXCR4 expression (pooled HR = 5.82, 95% CI = 3.01–11.25, P < 0.0001) and CD44 expression (pooled HR = 4.49, 95% CI = 2.12–9.53, P < 0.0001) predicted poor DFS. One study showed [35] no significant effect of CD105 in DFS (HR = 2.82, 95% CI = 0.99–9.06, P =0.05). High CD133 expression did not correlate with DFS (HR = 1.26, 95% CI = 0.55–2.87, P =0.58) in 1 study [14]. We did not perform a subgroup for the association between individual CSCs markers and DFS because the eligible studies were limited. Only one article [24] reported PFS for metastatic RCC patients. The study showed that high CXCR4 expression predicted sunitinib responsiveness on PFS (HR = 1.26, 95% CI = 0.55–2.87, P =0.04).

### Sensitivity analysis

In order to gauge the stability of the results, sensitivity analysis was performed by assessing the potential impact of individual studies on pooled data. As shown in Table 3, the combined HR of the association of CD44 expression with CSS was affected and heterogeneity was observed again by omitting 1 study [34]. However, the remaining pooled HR was not significantly altered.

**Table 3:** The influence of individual study on the pooled estimate for outcomes

Outcomes	Study omitted	Years	HR	95%CI	Heterogeneity	
					I^2^(%)	P value
OS(CXCR4)	None		2.57	1.95-3.40	42%	0.10
	An et al (cohort 1)	2014	2.48	1.85-3.34	48%	0.07
	An et al (cohort 2)	2014	2.54	1.89-3.41	50%	0.06
	D’Alterio et al	2012	3.43	2.43-4.83	0%	0.53
	Gassenmaier et al	2012	2.51	1.88-3.34	48%	0.07
	Huang et al	2014	2.52	1.90-3.34	47%	0.08
	Li et al	2011	2.42	1.80-3.26	44%	0.10
	Li et al	2013	2.65	1.96-3.60	49%	0.06
	Wang et al	2012	2.38	1.78-3.17	27%	0.22
OS(CD44)	None		1.58	1.14-2.18	23%	0.25
	Bamias et al	2003	1.77	1.24-2.53	9%	0.36
	Jeong et al	2012	1.42	1.01-2.00	0%	0.51
	Lucin et al	2004	1.50	1.07-2.09	23%	0.26
	Mikami et al	2014	1.58	1.14-2.20	36%	0.17
	Qin et al	2014	1.52	1.08-2.11	29%	0.22
	Tawfik et al	2007	1.70	1.18-2.45	30%	0.21
	Zhang et al	2013	1.64	1.11-2.41	35%	0.17
CSS(CXCR4)	None		1.97	1.50-2.59	0%	0.45
	Chen et al	2014	1.91	1.44-2.52	0%	0.46
	Li et al	2013	2.00	1.27-3.14	14%	0.28
	Staller et al	2003	2.92	1.43-5.98	0%	0.63
CSS(CD44)	None		2.58	1.27-5.23	47%	0.13
	Costa et al	2012	3.31	1.55-7.07	37%	0.20
	Jeong et al	2012	2.01	1.24-3.27	0%	0.37
	Rioux-Leclercq et al	2001	3.00	0.91-9.96	63%	0.07
	Yildiz et al	2004	2.25	1.39-5.99	62%	0.07
DFS(CXCR4)	None		5.82	3.01-11.25	0%	0.50
	D’Alterio et al	2010	7.76	3.43-17.55	0%	0.88
	Huang et al	2014	5.55	2.41-12.77	26%	0.24
	Wang et al	2012	4.18	1.63-10.69	0%	0.50
DFS(CD44)	None		4.49	2.12-9.53	44%	0.17
	Daniel et al	2001	4.73	1.54-14.52	71%	0.06
	Jeong et al	2012	3.10	1.79-5.36	0%	0.54
	Paradis et al	1999	7.29	3.10-17.16	0%	0.46

### Publication bias

**Figure 5:**
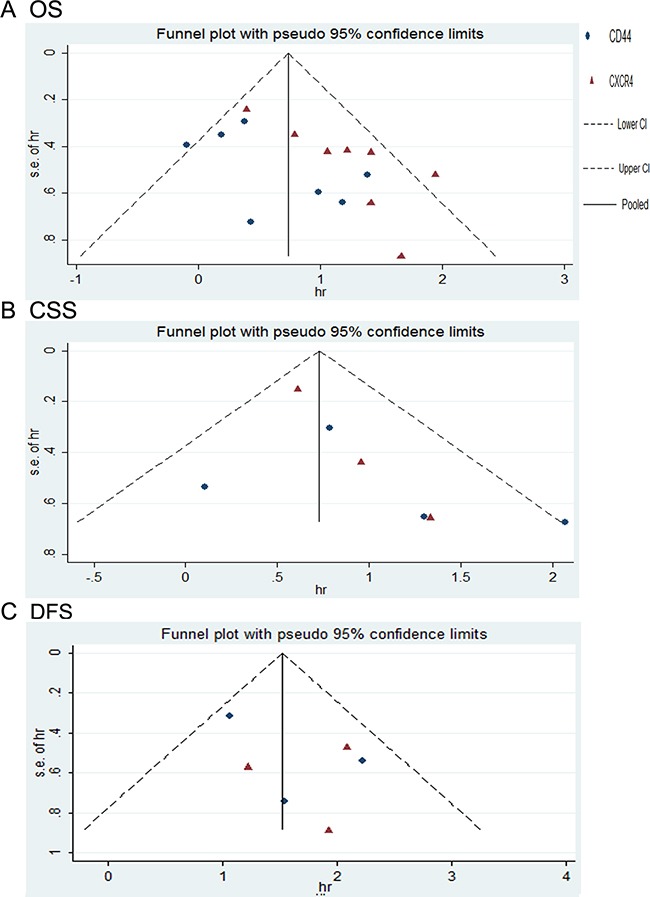
funnel plot for publication bias

Publication bias analysis of the studies was performed to test the reliability of the results. As shown in Figure 5, the funnel plots showed evidence for symmetry in CSS and DFS, but not in OS studies, suggesting that a publication bias about OS possibly existed. Then, Begg's and Egger's tests were conducted to more precisely assess the bias. As shown in Table 4, especially studies concerning CXCR4 expression and OS showed publication bias as analyzed by Egger's test (t -value =3.95, 95% CI =1.17-4.97, P =0.01) and Begg's test (P =0.03).

**Table 4:** Publication bias was determined for outcomes by begg and egger tests

Outcomes	Marker	Begg's test		Egger's test	
		P value	t-value	95%CI	P value
OS	CXCR4+CD44	0.09	2.75	0.51-4.27	0.02
OS	CXCR4	0.03	3.95	1.17-4.97	0.01
OS	CD44	0.55	1.65	−1.15-5.30	0.16
CSS	CXCR4+CD44	0.07	1.75	−0.55-2.91	0.14
CSS	CXCR4	0.30	10.75	−0.24-2.92	0.06
CSS	CD44	0.31	0.68	−8.09-11.3	0.57
DFS	CXCR4+CD44	0.71	1.25	−1.93-5.10	0.28
DFS	CXCR4	1.00	−0.16	−36.15-35.27	0.90
DFS	CD44	1.00	1.00	−26.44-30.93	0.50

## DISCUSSION

Although treatments for RCC have recently developed rapidly, including introduction of tyrosine kinase inhibitors (TKIs) and mTOR kinase inhibitors [42, 43], complete responses are rare. Thus, RCC still remains one of the deadliest forms of cancer and has a poor clinical outcome with recurrence or incomplete resection. RCC is characterized by a wide variation in prognosis. Biomolecular markers offer potential for additional information in cancer prognostic and predictive values. The conventional prognostic variables such as staging or grading cannot well predict clinical outcome on an individual basis [18]. From a clinical perspective, identifying new biomarkers for prognosis to guide surveillance is important and urgent.

Accumulated evidence shows that cancer can be considered as a stem cell disease [44]. CSCs, which comprise a small subpopulation of cancer cells, exhibit self-renewal ability and cancer-propagating capacity [45, 46]. The concept of contribution of CSCs to cancer initiation and therapeutic resistance is widely accepted, so a better understanding of the characteristics of CSCs will provide valuable therapeutic and prognostic targets for clinical practice. Recently, relevant markers of CSCs have been found to be independent prognostic factors for various cancers [47, 48]. While some studies have revealed that CSCs markers can be associated with RCC prognosis, others have not [31, 38, 40]. In our meta-analysis, we have attempted to resolve the conflicting data and thus to quantitatively estimate the prognostic value of CSCs markers in RCC patients.

This meta-analysis of 25 studies, including 2673 participants, indicated that adverse prognostic effects of CSCs markers on OS and DFS. The pooled data suggest that CSCs markers could be used as indicators of RCC outcome. A body of evidence indicates that CSCs can facilitate renal cancer cell growth, invasion and metastasis [26, 49], which may partially explain the association of CSCs markers expression with clinical outcome. In the stratified analysis by individual CSCs markers, combined HR showed that high expression of CXCR4 predicted poor prognosis of OS, especially in Asia, as well as CSS and DFS. One of reasonable explanation might be the stromal derived factor-1 (SDF-1/CXCR4) axis hypothesis. One study indicated that SDF-1, via interaction with CXCR4, contributed to RCC metastatic potential [50]. CD44, as a multifunctional cell surface adhesion molecule, has been identified as a marker of RCC CSCs. Another study found that an activated TNF-a/CD44 axis facilitates progression of RCC by enhancing epithelial-mesenchymal transition (EMT) [31]. Consistent with the previous report, our study suggests that high expression of CD44 significantly correlate with unfavorable OS, CSS and DFS. There are a few references about the relationship between CD133 and CD105 expression and RCC clinical outcome [14, 29, 35, 40]. The pooled HR suggests CD133 to be an independent favorable prognostic factor for CSS. CD133 was one of the most commonly used CSCs markers, and numerous studies indicated CD133 over-expression in cancer patients exhibited a poor prognosis [51, 52]. Considering that the sample sizes are relatively limited, these results need to be cautiously interpreted. Currently, CSCs modulators have been moved from theoretical basic research into preclinical and early clinical trials. CXCR4 inhibitor AMD3100 facilitates anti-angiogenic agents sunitinib and sorafenib anticancer effects via blockade of CXCR4^+^ RCC CSCs [11]. Also, it has been reported that IL-15 treatment of RCC CD105^+^CSCs could suppress cancer progression [10]. Since the association of CSCs markers with metastatic RCC survival (PFS) is scarce in literature, we did not reassess the correlation. Further investigation of the prognostic value of CSCs markers in metastatic RCC should be designed.

Several potential limitations should be acknowledged and some results need to be interpreted cautiously. The number of eligible studies was relatively small, especially in assessing the association of CD133 and CD105 with RCC prognosis, thus reducing the power of the results. The total sample sizes were relatively limited, which might lead to an erroneous conclusion. All of the enrolled studies were retrospective, making them more susceptible to information and selection biases. This study was constrained to articles published in English, which might contribute to selection bias. Moreover, for studies that did not provide HR and 95% CI directly, we evaluated and calculated the HRs via survival curves. This method might reduce the credibility of the results. Additionally, publication bias existed for OS, thus inflating the estimate for the association of CXCR4 with poor prognosis. The quality of included studies was assessed by NOS. We found heterogeneity may come from low-quality studies according to the results of subgroup analysis. Furthermore, the variations of the characteristics of patients and the various detecting antibodies against CSCs markers might have caused inherent heterogeneity within studies.

In conclusion, despite certain limitations, the present results provide some evidence on the prognostic value of CSCs markers in RCC. The presence of CSCs is associated with a poor clinical outcome. High CXCR4 and CD44 expression predicts a worse OS CSS and DFS. CD133 is an independent favorable prognostic factor for CSS. CSCs markers may potentially serve as prognostic stratification markers and novel potential therapeutic targets for RCC. Further large-scale and standard cohort studies are required for confirmation.

## MATERIALS AND METHODS

### Literature search strategy

This study adhered to the PRISMA guidelines [53]. A comprehensive literature search was conducted using PubMed and EMBASE databases from inception to 1 February 2016 in order to identify published articles assessing the prognostic value of CSCs markers in RCC. The terms for synonyms: “renal or kidney,” “cancer or tumor or carcinoma,” “CD44,” “Endoglin or CD105,” “Prominin-1or CD133,” “CXCR4,” “prognosis or survival or outcome,” were applied during the search. Searches were limited to publications in English. The PubMed and EMBASE databases search options were summarized in “Appendix”. The bibliographies of articles were also checked for additional eligible studies. Results and any disagreements were double-checked and arbitrated by a second reviewer.

### Study selection

All candidate articles initially were screened by titles and/or abstracts using the following inclusion criteria: 1) patients with RCC diagnosis which were pathologically confirmed; 2) RCC CSCs relevant markers (CD133, CXCR4, CD44, and CD105) expression was examined by immunohistochemistry (IHC) or polymerase chain reaction (PCR); 3) studies evaluated the association of CSCs markers expression with RCC survival outcomes [(disease-free survival (DFS), overall survival (OS), cancer-specific survival (CSS), progression-free survival (PFS)], hazard ratios (HR) with 95% confidence intervals (CI); 4) sample size≥20 cases; 5) If multiple articles were reported by the same cohorts, only the most complete paper was included. Non-research articles or studies that were focused on animal or human cell lines or papers lacking information on RCC prognosis were excluded.

### Data extraction

All eligible studies were identified by two independent investigators. The following data were extracted: general information (first author's surname, year of publication, country of origin), study population characteristics (patients number, age, and sex), follow-up data (median/mean follow-up duration, OS, CSS, PFS, and DFS with corresponding 95% CI), CSCs markers expression data (assessment method and cut-off value). If the HR and 95% CI were not displayed directly, they were estimated from Kaplan–Meier curves as reported by Tierney et al [54].

### Qualitative assessment

The quality of each of the eligible studies was assessed independently by 2 investigators using the Newcastle–Ottawa Quality Assessment Scale (NOS) for cohort studies (Supplemental Table S2). Briefly, the scale uses a star system to indicate the quality of each study (Supplemental Table S3). Studies that received a score of ≥7 stars were considered to be of high quality [55].

### Statistical analysis

HR values with 95% CI for OS, CSS, PFS, and DFS according to the expression of CSCs markers were pooled. In this study, a combined HR>1 reflected a worse prognosis for high CSCs markers expression patents, while a pooled HR<1 indicated a better survival. Z test for pooled HR and a P-value < 0.05, or no overlap of the 95% CI with 1 was considered statistically significant. The fixed-effects model (FEM) or the random-effects model (REM) was used to evaluate heterogeneity [56], which was verified using chi-square-based Cochran Q-test. The I^2^ value implied the degree of heterogeneity. The REM was used for data showing statistically significant heterogeneity if P <0.05 and/or I^2^>50%, otherwise, FEM was applied. Subgroup analysis was performed to explore the potential sources of heterogeneity. Potential publication bias was assessed by funnel plot and precisely evaluated by Egger's and Begg's tests [57, 58]. The robustness of the pooled data was examined by sensitivity analysis. Kaplan–Meier curves were read by Engauge Digitizer version 4.1(http://sourceforge.net). Stata 10.0 (Stata Corporation, College Station, TX, USA) and Review Manager 5.2 (Cochrane Collaboration, London, UK) were used to statistical analyses in this study. All statistical tests were two-sided.

## SUPPLEMENTARY TABLES


