# Supplementary material for: Cancer stem cell markers predict a poor prognosis in renal cell carcinoma: a meta-analysis
Source: Oncotarget. 2016 Aug 29;7(40):65862–75. doi: 10.18632/oncotarget.11672 (PMC5323198; doi:10.18632/oncotarget.11672)
Supplement: Supplementary file 1 [file oncotarget-07-65862-s001.pdf]

Cancer stem cell markers predict a poor prognosis in renal cell carcinoma: a meta-analysis

SUPPLEMENTARY TABLES

Supplementary Table S1: List of adjustment factors employed in all studies included in current meta-analysis

Author	Year	Adjustment for covariates
D'Alterio et al	2010	Age, Gender, Clinical presentation, Stage, Fuhrman grade
Huang et al	2014	NR
Li et al	2001	NR
D'Alterio et al	2012	Age, Gender, Fuhrman grade, Response to sunitinib
Li et al	2013	Age,pT,pN,pM, Fuhrman grade, ECOG performance status, Necrosis.
Wang et al	2012	Age, Gender, Tumor size, Lymph nodal status, Histologic variant, Fuhrman grade.
Chen et al	2010	Stage, Fuhrman grade.
Staller et al	2003	NR
An et al	2014	Tumor size, Stage, Fuhrman grade, ECOG performance status, Necrosis.
Gassenmaier et al	2012	Tumor stage, Lymph node metastasis, Metastasis, Grade.
Saroufim et al	2014	Age, Gender, Stage, Leibovich risk.
Zhang et al	2013	Age, Vasculogenic mimicry, Fuhrman grade, Microvessel density.
Kim et al	2012	TNM stage, Histologic subtype, Fuhrman grade.
Costa et al	2011	Clinical stage, Tumor size, Fuhrman grade, MVI, Necrosis, Metastasis, ECOG performance status.
Mikami et al	2014	Tumor stage, Lymph node metastasis, Distant metastasis, Fuhrman grade, TNF-expression.
Qin et al	2014	Age, Gender, Stage, Fuhrman grade.
Costa et al	2012	Clinical stage, Tumor size, Fuhrman grade, MVI, Necrosis, Metastasis, ECOG performance status.
Tawfik et al	2007	Stage, Fuhrman grade.
Lucin et al	2011	TNM stage,Fuhrman grade.
Yildiz et al	2004	NR
Bamias et al	2012	CA 125, Stage, Grade
Rioux-Leclercq et al	2014	Stage, Tumor size, Fuhrman grade,MVD.Ki-67.
Daniel et al	2001	Stage, Tumor size, Fuhrman grade.
Paradis et al	1999	Tumor size, Fuhrman grade.
Jeong et al	2012	Tumor size, Age, Gender, ECOG performance status, Stage, Fuhrman grade.

Notes: NR: Not reported; MVI: Microvascular invasion;

Supplementary Table S2: newcastle – ottawa quality assessment scale

Item
**Selection**
(1) Representativeness of the exposed cohort
(a) Truly representative of the average 'renal cell carcinoma patients' in the community (1 star)
(b) Somewhat representative of the average 'renal cell carcinoma patients' in the community (1 star)
(c) Selected group of users (e.g. nurses, volunteers)
(d) No description of the derivation of the cohort
(2) Selection of the non-exposed cohort
(a) Drawn from the same community as the exposed cohort (1 star)
(b) Drawn from a different source
(c) No description of the derivation of the non-exposed cohort
(3) Ascertainment of exposure (Proof of renal cell carcinoma and CXCR4,CD133,CD44,CD105 measurement)
(a) Secure record (eg. surgical records) (1 star)
(b) Structured interview (1 star)
(c) Written self-report
(d) No description
(4) Demonstration that outcome of interest was not present at start of study
(a) Yes (1 star)
(b) No
**Comparability**
(1) Comparability of cohorts on the basis of the design or analysis
(a) Study controls for 'metastasis or recurrence' (1 star)
(b) Study controls for any additional factor (1 star) (Age, stage, grade etc.)
**Outcome**
(1) Assessment of outcome (Death or recurrence)
(a) Independent blind assessment (1 star)
(b) Record linkage (1 star)
(c) Self-report
(d) No description
(2) Was follow-up long enough for outcomes to occur?
(a) Yes ('3 years') (1 star)
(b) No
(3) Adequacy of follow-up of cohorts
(a) Complete follow-up – all subjects accounted for (1 star)
(b) Subjects lost to follow-up unlikely to introduce bias – small number lost '(25%)' or description provided of those lost (1 star)
(c) Follow-up rate '<75%' and no description of those lost
(d) No statement

Notes: *a study can be awarded a maximum of one point for each numbered item within the "selection" and "Outcome" categories, and a maximum of two points can be given for "comparability".

Supplementary Table S3: Methodological quality of all studies based on the Newcastle-Ottawa scale for assessing the quality of cohort trials

Author	Represen-tativeness of exposed cohort	Selection of non-exposed cohort	Assessment of exposure	Outcome not present at start of study	Comparability based on the design or analysis	Assessment of outcome	Follow-up long enough for outcomes	Adequacy of follow-up	Total score
D'Alterio et al 2010	1	1	1	1	1	1	1	1	8
Huang et al 2014	1	1	1	1	0	1	0	0	5
Li et al 2011	1	1	1	1	1	1	1	1	8
D'Alterio et al 2012	1	1	1	1	1	1	0	0	6
Li et al 2013	1	1	1	1	0	1	1	1	7
Wang et al 2012	1	1	1	1	1	1	1	0	7
Chen et al 2014	1	1	1	1	0	1	0	0	5
Staller et al 2003	1	1	1	1	0	1	0	0	5
An et al 2014	1	1	1	1	2	1	1	1	9
Gassenmaier et al 2012	1	1	1	1	1	1	0	0	6
Saroufim et al 2014	1	1	1	1	1	1	1	1	8
Zhang et al 2013	1	1	1	1	1	1	1	1	8
Kim et al 2012	1	1	1	1	0	1	1	1	7
Costa et al 2011	1	1	1	1	1	1	0	0	6
Mikami et al 2014	1	1	1	1	2	1	0	1	8
Qin et al 2014	1	1	1	1	2	1	1	0	9
Costa et al 2012	1	1	1	1	1	1	0	0	6
Tawfik et al 2007	1	1	1	1	0	1	0	0	5
Lucin et al 2004	1	1	1	1	1	1	1	0	7
Yildiz et al 2004	1	1	1	1	0	1	1	1	7
Bamias et al 2003	1	1	1	1	0	1	0	0	5
Rioux-Leclercq et al 2001	1	1	1	1	0	1	1	1	7
Daniel et al 2001	1	1	1	1	1	1	1	1	8
Paradis et al 1999	1	1	1	1	0	1	1	1	7
Jeong et al 2012	1	1	1	1	1	1	0	0	6

APPENDIX

SEARCH STRATEGY

Pubmed

#1.Search:((((((((((((((((("Kidney Neoplasms" [Mesh]) OR Kidney Neoplasms [Title/Abstract]) OR Kidney Neoplasm [Title/Abstract]) OR Neoplasm, Kidney [Title/Abstract]) OR Renal Neoplasms [Title/Abstract]) OR Neoplasm, Renal [Title/Abstract]) OR Neoplasms, Renal [Title/Abstract]) OR Renal Neoplasm [Title/Abstract]) OR Neoplasms, Kidney [Title/Abstract]) OR Cancer of Kidney [Title/Abstract]) OR Kidney Cancers [Title/Abstract]) OR Renal Cancer [Title/Abstract]) OR Cancer, Renal [Title/Abstract]) OR Cancers, Renal [Title/Abstract]) OR Renal Cancers [Title/Abstract]) OR Cancer of the Kidney [Title/Abstract]) OR Kidney Cancer[Title/Abstract]) OR Cancer, Kidney [Title/Abstract]) OR Cancers, Kidney [Title/Abstract]= 108909.

#2. Search:(((((((((Receptors, CXCR4 [MeSH Terms]) OR CXCR4 Receptor [Title/Abstract]) OR Receptor, CXCR4 [Title/Abstract]) OR Receptor, LESTR [Title/Abstract]) OR Fusin [Title/Abstract]) OR LESTR Receptor [Title/Abstract]) OR Leukocyte-Derived Seven-Transmembrane Domain Receptor [Title/Abstract]) OR Leukocyte Derived Seven Transmembrane Domain Receptor [Title/Abstract]) OR CXC Chemokine Receptor 4 [Title/Abstract]) OR CXCR4 Receptors [Title/Abstract]10045.

#3. Search:(((((((((((Antigens, CD44[MeSH Terms]) OR Hyaluronan-Binding Protein[Title/Abstract]) OR Hyaluronan-Binding Protein[Title/Abstract]) OR CD44 Antigen[Title/Abstract]) OR Antigen, CD44[Title/Abstract]) OR Receptors, Hyaluronan [Title/Abstract]) OR Hyaluronan Receptor [Title/Abstract]) OR Receptor, Hyaluronan [Title/Abstract]) OR Hyaluronan Receptors [Title/Abstract]) OR Hyaluronic Acid Binding Protein [Title/Abstract]) OR CD44 Antigens [Title/Abstract]) OR CD44 [Title/Abstract] 13976

#4. Search:((((((((((((((((AC133 antigen[MeSH Terms]) OR AC133-1 antigen [Title/Abstract]) OR CD133 antigen [Title/Abstract]) OR fudenine [Title/Abstract]) OR prominin-1 [Title/Abstract]) OR prominin-like PROML1 [Title/Abstract]) OR PROML1 [Title/Abstract]) OR AC 133 antigen[Title/Abstract]) OR prominin [Title/Abstract]) OR AC141 antigen [Title/Abstract]) OR AC133-2 antigen [Title/Abstract]) OR PROM1 protein, human [Title/Abstract]) OR prominin 1 protein, human [Title/Abstract]) OR CD133 protein, human [Title/Abstract]) OR AC133 antigen, human [Title/Abstract]) OR AC133 protein, human [Title/Abstract]) OR CD133 [Title/Abstract] 4600.

#5.Search:((((((((((((ENG protein, human [MeSH Terms]) OR endoglin protein, human [Title/Abstract]) OR Osler-Rendu-Weber syndrome 1 protein, human [Title/Abstract]) OR endoglin [Title/Abstract]) OR CD105 antigen, human [Title/Abstract]) OR CD105 [Title/Abstract]) OR CD105 antigen [Title/Abstract]) OR L-endoglin protein, human [Title/Abstract]) OR L-endoglin protein [Title/Abstract]) OR S-endoglin protein, human [Title/Abstract]) OR S-endoglin protein [Title/Abstract]) OR S-endoglin [Title/Abstract]) OR L-endoglin [Title/Abstract] 9676.

#6.Search:((((((((((((((("Prognosis" [Mesh]) OR Prognosis [Title/Abstract]) OR Prognoses [Title/Abstract]) OR Prognostic [Title/Abstract]) OR Outcome [Title/Abstract]) OR Survival [Title/Abstract]) OR Overall survival [Title/Abstract]) OR OS [Title/Abstract]) OR Cancer-specific survival [Title/Abstract]) OR CSS [Title/Abstract]) OR Progression-free survival [Title/Abstract]) OR PFS [Title/Abstract]) OR Disease-free survival [Title/Abstract]) OR DFS [Title/Abstract]) OR Mortality [Title/Abstract]) OR Recurrence [Title/Abstract] 2694987.

#7: #2 or #3 or #5: 36536.

#8: #1 and #6 and #7 119.

Embase

#1.'Kidney Cancer'/exp OR 'Kidney Cancer':ab,ti OR 'Neoplasm, Kidney':ab,ti OR 'Kidney Neoplasm':ab,ti OR 'Renal Neoplasms':ab,ti OR 'Neoplasm, Renal':ab,ti OR 'Neoplasm, Renal':ab,ti OR 'Neoplasms, Renal':ab,ti OR 'Renal Neoplasm':ab,ti OR 'Neoplasms, Kidney':ab,ti OR ' Cancer of Kidney ':ab,ti OR 'Kidney Cancers':ab,ti OR 'Renal Cancer':ab,ti OR 'Cancer, Renal':ab,ti OR 'Cancers, Renal':ab,ti OR 'Renal Cancers':ab,ti OR 'Cancer of the Kidney':ab,ti OR 'Kidney Cancer':ab,ti OR 'Cancer, Kidney ':ab,ti OR 'Cancers, Kidney':ab,ti 83458.

#2.'chemokine receptor CXCR4'/exp OR 'CXCR4 Receptor' OR 'Receptor, CXCR4':ab,ti OR ' Receptor, LESTR':ab,ti OR 'Fusin':ab,ti OR 'LESTR Receptor':ab,ti OR 'Leukocyte-Derived Seven-Transmembrane Domain Receptor':ab,ti OR 'Leukocyte Derived Seven Transmembrane Domain Receptor':ab,ti OR 'Chemokine Receptor CXC4':ab,ti OR 'CXCR4 Receptors':ab,ti OR 'CXCR4':ab,ti 17601.

#3.'Antigens, CD44'/exp OR 'Antigens, CD44' OR 'Hyaluronan-Binding Protein':ab,ti OR 'Hyaluronan-Binding Protein':ab,ti OR 'CD44 Antigen':ab,ti OR 'Antigen, CD44':ab,ti OR 'CD44':ab,ti OR 'Receptors, Hyaluronan':ab,ti OR 'Hyaluronan Receptor' OR 'Receptor, Hyaluronan':ab,ti OR 'Hyaluronan Receptors':ab,ti OR 'Hyaluronic Acid Binding Protein':ab,ti OR 'CD44 Antigens ':ab,ti 20930.

#4.’CD133 antigen’/exp OR ‘CD133 antigen’:ab,ti OR ‘AC133 antigen’:ab,ti OR ‘Hyaluronan-Binding Protein’:ab,ti OR ‘AC133-1 antigen’:ab,ti OR ‘fudenine’:ab,ti OR ‘prominin-1’:ab,ti OR ‘prominin-like PROML1’:ab,ti OR ‘PROML1’:ab,ti OR ‘AC 133 antigen’:ab,ti OR ‘prominin’:ab,ti OR ‘AC141 antigen’:ab,ti OR ‘AC133-2 antigen’:ab,ti OR ‘PROM1 protein, human’:ab,ti OR ‘AC133 antigen, human’:ab,ti OR ‘AC133 protein, human’:ab,ti OR ‘CD133’:ab,ti 9394.

#5.’endoglin’/exp OR ‘endoglin’:ab,ti OR ‘ENG protein, human’:ab,ti OR ‘endoglin protein, human’:ab,ti OR ‘Osler-Rendu-Weber syndrome 1 protein, human’:ab,ti OR ‘CD105 antigen, human’:ab,ti OR ‘CD105 antigen’:ab,ti OR ‘CD105’:ab,ti OR ‘L-endoglin protein, human’:ab,ti OR ‘L-endoglin protein’:ab,ti OR ‘S-endoglin protein, human’:ab,ti OR ‘S-endoglin protein’:ab,ti OR ‘S-endoglin’:ab,ti OR ‘L-endoglin’:ab,ti 7476.

#6.’prognosis’/exp OR ‘prognosis’ OR ‘prognosis’:ab,ti OR ‘prognoses’:ab,ti OR ‘prognostic’:ab,ti OR ‘outcome’:ab,ti OR ‘survival’:ab,ti OR ‘overall survival’:ab,ti OR os OR ‘cancer-specific survival’:ab,ti OR ‘css’:ab,ti OR ‘progression-free survival’:ab,ti OR ‘pfs’:ab,ti OR ‘disease-free survival’:ab,ti OR ‘dfs’:ab,ti OR ‘mortality’:ab,ti OR ‘recurrence’:ab,ti 2892062.

7. #2 OR #3 OR #4 OR #5 50490.

8. #1 AND #6 AND #7 267.
